# The regulatory roles of circular RNAs *via* autophagy in ischemic stroke

**DOI:** 10.3389/fneur.2022.963508

**Published:** 2022-10-18

**Authors:** Xiaoqin Li, Lingfei Li, Xiaoli Si, Zheng Zhang, Zhumei Ni, Yongji Zhou, Keqin Liu, Wenqing Xia, Yuyao Zhang, Xin Gu, Jinyu Huang, Congguo Yin, Anwen Shao, Lin Jiang

**Affiliations:** ^1^The Fourth School of Clinical Medicine, Zhejiang Chinese Medical University, Hangzhou, China; ^2^Department of Neurology, Affiliated Hangzhou First People's Hospital, Zhejiang University School of Medicine, Hangzhou, China; ^3^Department of Neurology, The Second Affiliated Hospital, School of Medicine, Zhejiang University, Hangzhou, China; ^4^Department of Emergency, Affiliated Hangzhou First People's Hospital, Zhejiang University School of Medicine, Hangzhou, China; ^5^Department of Cardiology, Affiliated Hangzhou First People's Hospital, Zhejiang University School of Medicine, Hangzhou, China; ^6^Department of Neurosurgery, The Second Affiliated Hospital, School of Medicine, Zhejiang University, Hangzhou, China; ^7^Key Laboratory of Precise Treatment and Clinical Translational Research of Neurological Disease, Hangzhou, China

**Keywords:** ischemic stroke, autophagy, circRNA, biomarkers, therapeutic targets

## Abstract

Ischemic stroke (IS) is a severe disease with a high disability, recurrence, and mortality rates. Autophagy, a highly conserved process that degrades damaged or aging organelles and excess cellular components to maintain homeostasis, is activated during IS. It influences the blood–brain barrier integrity and regulates apoptosis. Circular RNAs (circRNAs) are novel non-coding RNAs involved in IS-induced autophagy and participate in various pathological processes following IS. In addition, they play a role in autophagy regulation. This review summarizes current evidence on the roles of autophagy and circRNA in IS and the potential mechanisms by which circRNAs regulate autophagy to influence IS injury. This review serves as a basis for the clinical application of circRNAs as novel biomarkers and therapeutic targets in the future.

## Introduction

Stroke is a leading cause of death and disability worldwide (1) and can be classified as ischemic stroke (IS) or hemorrhagic stroke (2). The major type is IS, accounting for 71% of cases (2). During IS, ischemia and hypoxia cause neuronal and glial anoxic depolarization (3), which increases extracellular levels of glutamate, leading to excess calcium influx and release of calcium from intracellular stores (4). Increased intracellular calcium contributes to neuronal nitric oxide synthase activation with consequent free radical production and the initiation of cell death processes, including apoptosis, necrosis, necroptosis, and autophagy. Current effective treatments for IS include restoration of blood flow through intravenous thrombolysis and neuroscientific intravascular recanalization, both of which reduce disability (2); however, these treatment methods are still limited owing to the limited time window, numerous contraindications (5), and high risk of hemorrhagic complications (6).

Autophagy is activated to varying degrees after IS to restore neuronal homeostasis (2, 7). Autophagy functions in IS by sequestering damaged or aged organelles, superfluous proteins, and cellular components into double membrane-bound vesicles, delivering cytoplasmic cargo to the lysosome, to which it subsequently fuses to form an autolysosome, finally leading to digestion and recycling (8). Autophagy presents a dual effect following ischemic insult. Mild to moderate induction of autophagy can be protective in IS (9), whereas an excessive increase in autophagic activity might be harmful owing to the cytosolic accumulation of autophagosomes and enhanced degradation of essential cellular components (10). Autophagy is divided into two groups according to the role it plays in IS: maladaptive and adaptive autophagy (11).

Circular RNAs are a novel type of non-coding RNAs (12) with a stable and evolutionally conserved covalent loop structure (13). Previous studies have demonstrated that circRNAs are often specifically expressed in tissue and developmental stages (14) and are highly expressed in the mammalian brain (15). CircRNAs are upregulated during neuronal differentiation and are highly enriched in synapses (16). The role of circRNAs has been identified in several human diseases, including neurological disorders, cardiovascular diseases, diabetes mellitus, chronic inflammatory diseases, and cancer (17–21). Interestingly, circRNAs function in ischemic brain injury (22–25); therefore, they are potential biomarkers for IS and may serve as new therapeutic targets.

## CircRNAs

### The biogenesis and functions of circRNAs

**Figure 1:**
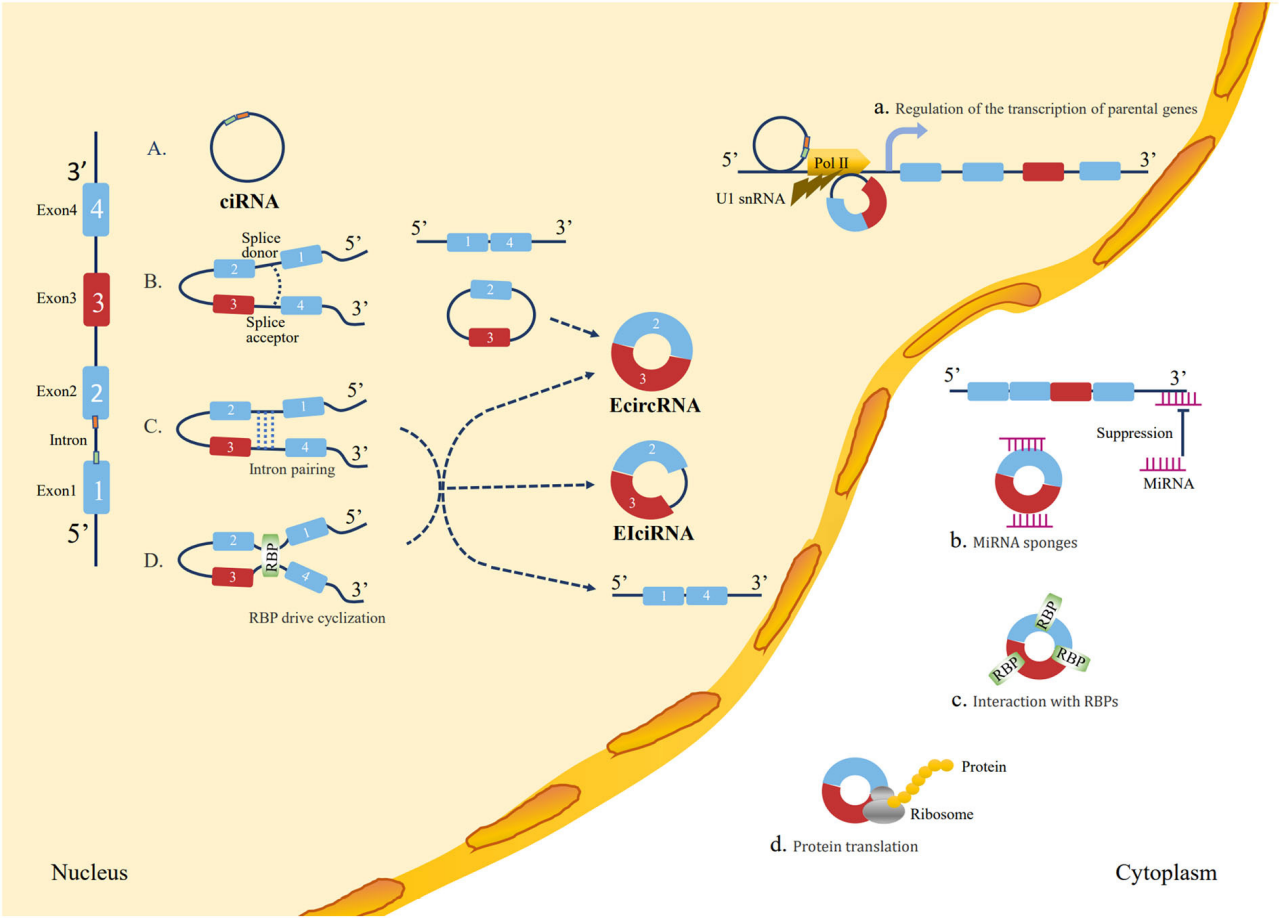
Loop formation mechanisms and biological functions of circRNAs. Loop formation mechanisms: **(A)** ciRNA formation: the elements near the splice site escape debranching stably so that the intron lariat is formed from the splicing reaction. **(B)** Lariat-driven circularization: the 5′ splice donor of exon 1 and the 3′ splice acceptor of exon 4 link up end-to-end by exon skipping and form an exon-containing lariat structure. Finally, the ecircRNA forms after introns are removed. **(C)** Intron pairing-driven circularization: direct base pairing of introns forms a circulation structure, thereby forming ecircRNA or EIciRNA after intron removal. **(D)** RBP-driven cyclization: RBPs bridge two flanking introns close together and then remove introns to form circRNAs. Biological functions: **(a)** Regulation of the transcription of parental genes: circRNAs play a regulatory role in the transcription of their parent coding genes. **(b)** Function as miRNA “sponge”: circRNAs contain a common miRNA response element (MRE) that can bind to miRNA and prevent them from interacting with mRNA. **(c)** Interaction with RBPs: circRNAs can bind to RBPs to regulate mRNA expression by altering the splicing pattern or mRNA stability. **(d)** Protein translation: circRNAs have coding potential and can be translated into proteins with ribosomes.

Circular RNAs are a typical class of non-coding RNAs with a covalent loop structure (13), which were first discovered in RNA viruses in 1979 (57) and were originally hypothesized to be a by-product of mis-splicing (58). Due to different splicing processes, circRNAs were divided into three main types, including exonic circRNAs (ecircRNAs), exon-intron circRNAs (EIciRNAs), and circular intronic RNAs (ciRNAs) (59). However, with advances in experimental technology, emerging evidence has indicated that circRNAs are implicated in the occurrence and development of many diseases, such as cancer (60), cardiovascular disorders (61), neurological disorders (62), metabolic disorders (63), and immunological disorders (64). The functions of circRNAs are as follows: acting as a sponge of miRNA or competitive endogenous RNAs (ceRNAs), interacting with RNA-binding proteins (RBPs) and mRNAs, regulating transcription or alternative splicing, and protein translation (65). The biogenesis and functions of circRNAs are illustrated in Figure 1.

### CircRNAs participating in pathological processes in ischemic stroke: Apoptosis, inflammation, oxidative stress, angiogenesis, and autophagy

Ischemic stroke is a complex multifactorial disease in which the cascade response to ischemia is not a single linear process but often involves parallel or cross-interacting mechanisms and events (66, 67). CircRNAs are involved in various mechanisms and events in the ischemic cascade through both the regulations of host genes at the transcriptional level and the ceRNA mechanisms (68), such as apoptosis, inflammation, oxidative stress, angiogenesis, and autophagy. We summarize the evidences of circRNA regulatory roles in these pathophysiological changes of ischemic stroke (Table 1). Yang et al. found that circTTC3 levels were increased in oxygen-glucose deprivation (OGD)-treated astrocytes and middle cerebral artery occlusion/reperfusion (MCAOR) mice (50). The depletion of circTTC3 can decrease cerebral infarction, brain edema, and apoptosis, and attenuates cerebral infarction *via* the miR-372-3p/TLR4 axis (50). Zhang et al. found that circ_USP36 levels were increased in the serum of atherosclerosis patients and in oxidized low-density lipoprotein (ox-LDL)-stimulated human umbilical vein endothelial cells (HUVECs) (32). Circ_USP36 overexpression can trigger apoptosis and inflammation *via* the miR-197-3p/ROBO1 axis to promote atherosclerosis, which may finally result in IS (32). Yang et al. found that circPHKA2 levels were decreased in the venous blood of acute IS patients and OGD-induced human brain microvascular endothelial cells (HBMECs) (37). Overexpression of circPHKA2 can inhibit apoptosis, endoplasmic reticulum (ER) stress, and oxidative stress in HBMECs after OGD *via* the miR-574-5p/SOD2 axis (37). Bai et al. found that circFUNDC1 levels were increased in OGD-treated HBMECs (44). CircFUNDC1 knockdown can promote OGD-blocked cell viability, migration, and angiogenesis of HBMECs by inhibiting phosphatase and tensin homolog (PTEN) by enriching miR-375 (44). Yang et al. found that circ-FoxO3 levels were increased in mouse models with MCAO/R (23). Circ-FoxO3 protected against OGD/R-stimulated endothelial barrier damage and tMCAO-induced BBB collapse in mice by upregulating autophagy (23). Yang et al. found that circSCMH1 levels were significantly decreased in the plasma of patients with acute ischemic stroke, and in the plasma and the peri-infarct cortex of photothrombotic stroke mice (55). CircSCMH1 overexpression contributes to functional recovery post-stroke by enhancing neuronal plasticity, while inhibiting glial activation and peripheral immune cell infiltration (55). Interestingly, a single circRNA can influence more than one pathological process. Circ_0003204 is increased in ox-LDL-induced HUVECs. Circ_0003204 silencing weakens ox-LDL-induced cell viability inhibition, apoptosis, inflammatory response, and oxidative stress, but facilitates proliferation, migration, and angiogenesis (29, 33).

**Table 1:** The evidence of CircRNA regulatory roles in the pathophysiological changes of ischemic stroke.

**Pathological process in IS**	**Cell line/treatment**	**Animal/disease model**	**DE CircRNA and expression**		**Regulating axis**	**Overexpression role**	**References**
**Evidences from basic research**							
Apoptosis	Arterial SMCs, HEK-293, iPSC	/	Circ_ANRIL	Up	PES1	Induce apoptosis	(28)
	Transfection of siRNA					Detrimental	
	HCtAECs and THP-1 cells	/	Circ_0003204	Up	MiR-188-3p/TRPC6	Induce apoptosis	(29)
	Ox-LDL					Detrimental	
	HUVECs	/	Circ_0003204	Up	MiR-942-5p/HDAC9	Induce apoptosis	(30)
	Ox-LDL					Detrimental	
	HUVECs	/	Circ_0003204	Up	MiR-491-5p/ICAM1	Induce apoptosis	(31)
	Ox-LDL					Detrimental	
	HUVECs	/	Circ_0003204	Up	MiR-197-3p/ROBO1	Induce apoptosis	(32)
	Ox-LDL					Detrimental	
	HUVECs	/	Circ_0003204	Up	Unknown	Induce apoptosis	(33)
	Ox-LDL					Detrimental	
	HBMEC-IM cells	/	Circ_0003423	Down	MiR-589-5p/TET2	Inhibit apoptosis	(34)
	Ox-LDL					Protective	
	HUVECs	/	Circ_0003423	Down	MiR-142-3p/Sirtuin 3, SOD2	Inhibit apoptosis	(35)
	Ox-LDL					Protective	
	mAS	Mouse	CircCTNNB1	Down	MiR-96-5p/SRB1	Inhibit apoptosis	(36)
	OGD/R	MCAO				Protective	
	HBMECs	/	CircPHKA2	Down	MiR-574-5p/SOD2	Inhibit apoptosis	(37)
	OGD					Protective	
	SK-N-SH	/	Circ_0000647	Up	MiR-126-5p/TRAF3	Induce apoptosis	(38)
	OGD/R					Detrimental	
	HBMVECs	/	Circ-Memo1	Up	MiR-17-5p/SOS1	Induce apoptosis	(39)
	H/R					Detrimental	
Apoptosis	HCN-2	/	CircDlgap4	Down	MiR-503-3p/NEGR1	Inhibit apoptosis	(40)
	OGD					Protective	
	HUVECs	Mouse	CircDlgap4	Down	MiR-143/HECTD1	Inhibit apoptosis	(41)
	I/R	I/R				Protective	
	Astrocytes	Rats	Circ 0025984	Down	MiR-143-3p/TET1/ORP150	Inhibit apoptosis	(22)
	OGD	MCAO				Protective	
	Brain-derived endothelial cells	Mouse	Circ_0072309	Down	MiR-100/mTOR	Inhibit apoptosis	(42)
	OGD	MCAO				Protective	
	HT22 cells	Mouse	CircCDC14A	Up	MiR-23a-3p/CXCL12	Induce apoptosis	(43)
	OGD/R	MCAO				Detrimental	
	HBMECs	/	CircFUNDC1	Up	MiR-375/PTEN	Induce apoptosis	(44)
	OGD					Detrimental	
	HBMECs	/	CircPHC3	Up	MiR-455-5p/TRAF3	Induce apoptosis	(45)
	OGD					Detrimental	
	RGCs	Mouse	CGLIS3	Up	MiR-203/unknown	Induce apoptosis	(46)
	OGD/R	tMCAO				Detrimental	
	HT22 cells	Mouse	Circ-HECTD1	Up	MicroRNA-133b/TRAF3	Inhibit apoptosis	(47)
	OGD	MCAO				Protective	
	HT22 cells	Mouse	Circ-HECTD1	Up	MiR-27a-3p/FSTL1	Inhibit apoptosis	(48)
	OGD/R	MCAO				Protective	
	/	Mouse	Circ_008018	Up	MiR-99a/Akt, GSK3β	Induce apoptosis	(49)
		MCAO				Detrimental	
	Astrocytes	Mouse	CircTTC3	Up	MiR-372-3p/TLR4	Induce apoptosis	(50)
	OGD	MCAO/R				Detrimental	
Oxidative Stress	mAS	Mouse	CircCTNNB1	Down	MiR-96-5p/SRB1	Inhibit oxidative stress	(36)
	OGD/R	MCAO				Protective	
	HBMECs	/	CircPHKA2	Down	MiR-574-5p/SOD2	Inhibit oxidative stress	(37)
	OGD					Protective	
	SK-N-SH	/	Circ_0000647	Up	MiR-126-5p/TRAF3	Induce oxidative stress	(38)
	OGD/R					Detrimental	
Oxidative Stress	HBMVECs	/	Circ-Memo1	Up	MiR-17-5p/SOS1	Induce oxidative stress	(39)
	H/R					Detrimental	
	HCtAECs and THP-1 cells	/	Circ_0003204	Up	MiR-188-3p/TRPC6	Induce oxidative stress	(29)
	Ox-LDL					Detrimental	
	N2a cells, neurons	Mouse	CircDlgap4	Down	AUF1/NRF2 mRNA	Inhibit oxidative stress	(51)
	OGD	tMCAO				Protective	
Inflammation	HBMECs	/	Circ_ANRIL	Up	MiR-622	Induce inflammation	(52)
	OGD/R					Detrimental	
	HCtAECs and THP-1 cells	/	Circ_0003204	Up	MiR-188-3p/TRPC6	Induce inflammation	(29)
	Ox-LDL					Detrimental	
	HUVECs	/	Circ_0003204	Up	MiR-942-5p/HDAC9	Induce inflammation	(30)
	Ox-LDL					Detrimental	
	HUVECs	/	Circ_0003204	Up	MiR-491-5p/ICAM1	Induce inflammation	(31)
	Ox-LDL					Detrimental	
	HUVECs	/	Circ_0003204	Up	MiR-197-3p/ROBO1	Induce inflammation	(32)
	Ox-LDL					Detrimental	
	mAS	Mouse	CircCTNNB1	Down	MiR-96-5p/SRB1	Inhibit inflammation	(36)
	OGD/R	MCAO				Protective	
	SK-N-SH	/	Circ_0000647	Up	MiR-126-5p/TRAF3	Induce inflammation	(38)
	OGD/R					Detrimental	
	HBMVECs	/	Circ-Memo1	Up	MiR-17-5p/SOS1	Induce inflammation	(39)
	H/R					Detrimental	
	HCN-2	/	CircDlgap4	Down	MiR-503-3p/NEGR1	Inhibit inflammation	(40)
	OGD					Protective	
	HBMECs	/	Circ_0006768	Down	MiR-222-3p/VEZF1	Inhibit inflammation	(53)
	OGD/R					Protective	
Angiogenesis	HBMEC-IMs	/	Circ_0003423	Down	MiR-589-5p/TET2	Promote angiogenesis	(34)
	Ox-LDL					Protective	
	HUVECs	/	Circ_0003423	Down	MiR-142-3p/Sirtuin 3, SOD2	Promote angiogenesis	(35)
	OGD					Protective	
Angiogenesis	HBMECs	/	Circ_0006768	Down	MiR-222-3p/VEZF1	Promote angiogenesis	(53)
	OGD/R					Protective	
	HBMECs	/	CircFUNDC1	Up	MiR-375/PTEN	Inhibit angiogenesis	(44)
	OGD					Detrimental	
	HUVECs	/	Circ_0003204	Up	Unknown	Inhibit angiogenesis Detrimental	(33)
	Ox-LDL						
	HBMEC	/	CircPHKA2	Down	MiR-574-5p/SOD2	Promote angiogenesis	(37)
	OGD					Protective	
Autophagy	Astrocytes, A172cells	Mouse	CircHECTD1	Up	MiR142/TIPARP	Promote autophagy Detrimental	(25)
	OGD-R	tMCAO					
	Astrocytes	Mouse	CircSHOC2	Up	MiR-7670-3p/SIRT1	Inhibit autophagy	(24)
	OGD	MCAO				Protective	
	A172 and SK-N-AS	Mouse	Circ_0025984	Down	MiR-143-3p/TET1	Inhibit autophagy	(22)
	OGD	MCAO				Protective	
	BEnd.3 or HBMECs	Mouse	Circ-FoxO3	Up	MTOR and E2F1	Promote autophagy	(23)
	OGD/R	MCAO/R				Protective	
	HT22 cells	Mouse	Circ_016719	Up	MiR-29c/Map2k6	Promote autophagy	(26)
	OGD/R	tMCAO				Detrimental	
	Astrocytes	Mouse	CircAkap7	Down	MiR-155-5p/ATG12, NRF2	Promote autophagy	(27)
	OGD/R	tMCAO				Protective	
Other way	HASMCs, MASMCs	/	Circ_ACTA2	Up	MiR-548f-5p/α-SMA	Facilitate stress fiber formation and cell contraction	(54)
						Detrimental	
	Astrocytes, microglia	/	CircSCMH1	Down	MeCP2	Enhance neuronal plasticity, inhibit glial activation and peripheral immune cell infiltration	(55)
	OGD					Protective	
	NSCs	Mouse	CircHIPK2	Up	Smox	Reduce neuronal differentiation	(56)
	OGD/R	tMCAO				Detrimental	

PES1, Pescadillo homolog 1; TRPC6, transient receptor potential canonical channel 6; HDAC9, histone deacetylase 9; HUVECs, human umbilical vein endothelial cells; ICAM1, intercellular adhesion molecule 1; ROBO1, roundabout guidance receptor 1; ox-LDL, oxidized low-density lipoprotein; TET2, ten-eleven translocation 2; HBMECs, human brain microvascular endothelial cells; SOD2, superoxide dismutase 2; mAS, mouse astrocytes; OGD/R, oxygen and glucose deprivation/reoxygenation; MCAO, middle cerebral artery occlusion; SRB1, scavenger receptor class B type 1; OGD, oxygen and glucose deprivation; TRAF3, tumor necrosis factor receptor-associated factor 3; HBMVECs, human brain microvascular endothelial cells; H/R, hypoxia/reoxygenation; SOS1, Son of Sevenless 1; NEGR1, neuronal growth regulator 1; I/R, ischemia/reperfusion; TET1, ten-eleven translocation methylcytosine dioxygenase 1; ORP150, oxygen-regulated protein 150; mTOR, mechanistic target of rapamycin; CXCL12, C-X-C motif chemokine 12; PTEN, phosphatase and tensin homolog; FSTL1, follistatin-like 1; Akt, protein kinase B; GSK3β, glycogen synthase kinase 3β; TLR4, Toll-like receptor 4; TRPC6, transient receptor potential canonical channel 6; tMCAO, transient middle cerebral artery occlusion; NRF2, nuclear factor erythroid 2-related factor 2; HDAC9, histone deacetylase 9; ICAM1, intercellular adhesion molecule 1; NEGR1, neuronal growth regulator 1; VEZF1, vascular endothelial zinc finger 1; SIRT3, sirtuin 3; TIPARP, TCDD inducible poly(ADP-ribose) polymerase; SIRT1, sirtuin 1; E2F1, E2F transcription factor 1; Map2k6, Rac-MAPK kinase 6; ATG12, autophagy-related genes 12; HASMCs, human aortic smooth muscle cells; MASMCs, mouse aortic vascular smooth muscle cells; α-SMA, smooth muscle α-actin; MeCP2, methyl-CpG binding protein 2; Smox, spermine oxidase; SK-N-SH, Human neuroblastoma cell line; H/R, hypoxia/reoxygenation; HCN-2, human cortical neuronal cells-2; RGCs, retinal ganglion cells; HCtAECs, human carotid artery endothelial cells; NSCs, neural stem cells.

## Autophagy

### Overview of autophagy

Autophagy, a degradative and recycling process, is divided into three categories; namely, macroautophagy, microautophagy, and chaperone-mediated autophagy (CMA) (8). The process of autophagy involves the following four steps: initiation, nucleation, elongation, and fusion and degradation (7). With technological advances, the mechanisms and pathways of autophagy have been well-documented, such as the mammalian target of rapamycin (mTOR) pathway, the mitogen-activated protein kinase (MAPK) signaling pathway, and the hypoxia-inducible factor-1 (HIF-1) pathway, among others (69). A disturbance in cellular homeostasis leads to changes in autophagic processes.

### The role of autophagy in ischemic stroke

The obstruction of blood flow during a stroke can lead to complex pathophysiological processes (70) including oxidative stress, inflammation, BBB breakdown, calcium overload, excitatory toxicity, and (71) autophagy dysfunctions, leading to neurological disasters. Subsequent recanalization of blood flow (72) results in a secondary injury known as ischemia-reperfusion (I/R) injury.

**Figure 2:**
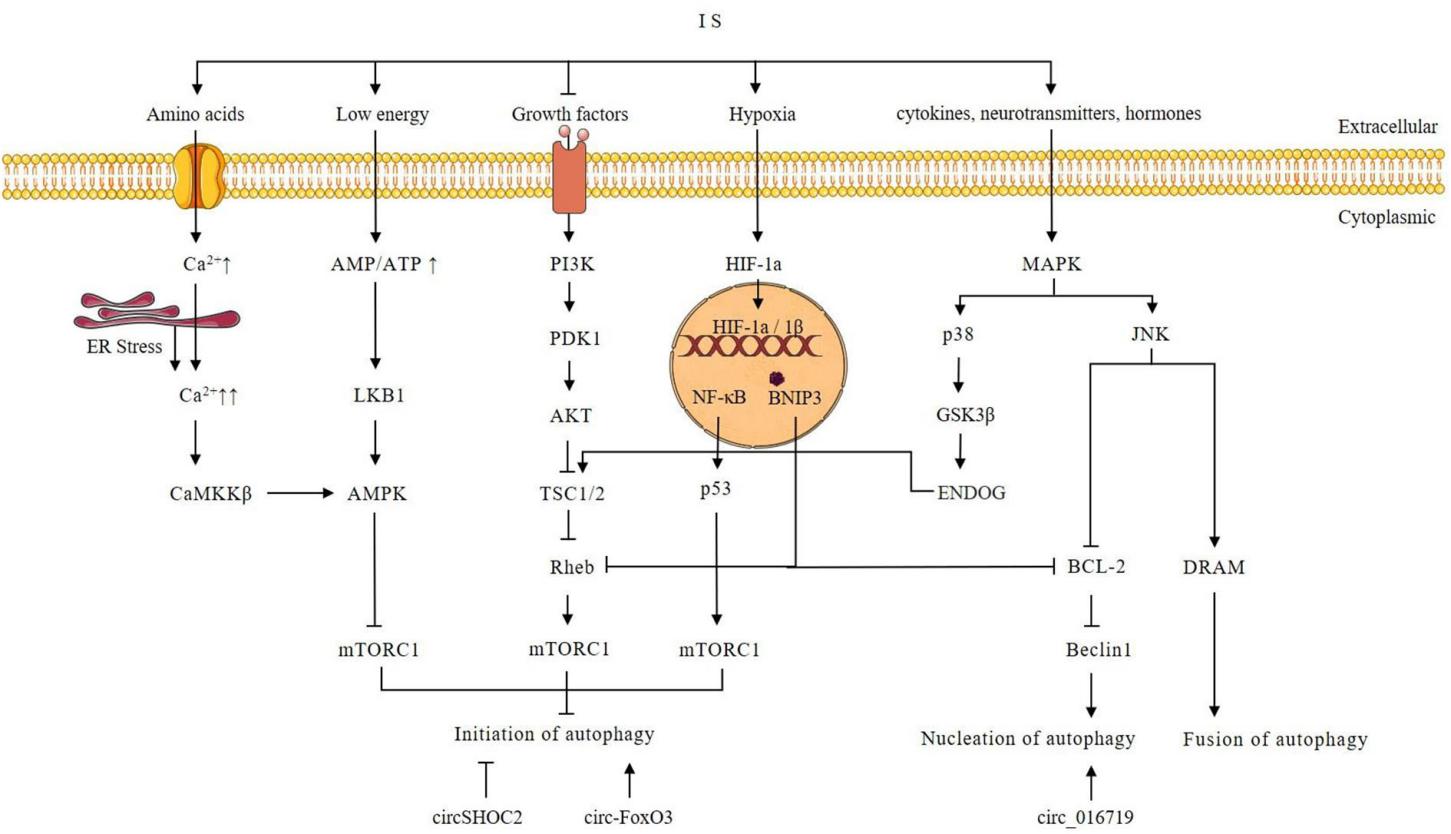
Possible autophagy signaling pathway after ischemic stroke and possible autophagy-regulating mechanism of circRNAs. Intracellular Ca^2+^ is increased during IS. Increased Ca^2+^ triggers ER stress and activates CaMKK, which in turn phosphorylates and activates AMPK. AMPK mediates the initiation of autophagy through the inhibition of mTORC1. Growth factors can activate PI3K. Then PI3K activates Akt through phosphorylation, and subsequently, the activated Akt directly phosphorylates and thereby inhibits TSC1/2. The Akt-dependent phosphorylation results in the dissociation of TSC1/2 from the lysosome, where Rheb is localized, promoting Rheb activation. Since GTP-bound Rheb is a potent mTORC1 activator, inhibition of TSC1/2 by AKT-dependent phosphorylation results in mTORC1 activation. Autophagy is inhibited by activated mTORC1. However, under conditions of growth factors or amino acid insufficiency during IS, mTORC1 activity is reduced and induces autophagy. Hypoxia caused by IS activates HIF-1α and induces autophagy through BNIP3 and p53. During IS, an increased AMP/ATP ratio activates LKB1 kinase, which in turn phosphorylates and activates AMPK. AMPK mediates the induction of autophagy through the inhibition of mTORC1. Activation of the p38 MAPK signaling pathway in IS phosphorylates GSK3β, which can subsequently activate ENDOG and TSC1/2. Activated TSC1/2 promotes the initiation of autophagy by inhibiting mTORC1. JNK activation modulates autophagy by promoting Bcl-2/Bcl-xL phosphorylation and upregulating DRAM. Beclin-1 interacts with Bcl2 through its unique BH3 pattern to form the Bcl2-Beclin-1 complex *via* promoting Beclin-vps34-p50 complex dissociation to inhibit autophagy. DRAM can promote autophagy by stimulating autophagosome-lysosome fusion. Overexpression of circSHOC2 inhibits the initiation of autophagy. Overexpression of circ-FoxO3 can promote the initiation of autophagy through inhibition of mTORC1. Overexpression of circ_016719 promotes nucleation of autophagy.

Autophagy is activated during IS and has a dual effect. Autophagy imbalance can aggravate brain damage (maladaptive autophagy), whereas proper autophagy (adaptive autophagy) helps to maintain brain metabolism (73, 74). Maladaptive autophagy is an excessive autophagic activity that accelerates the cytoplasmic accumulation of autophagosomes and the degradation of basic cellular components, triggering apoptosis and necrosis, and eventually causing neuronal cell death and aggravating neurological damage (11). In contrast, adaptive autophagy is a mild to moderate induction of autophagy that promotes neural cell survival (11). During IS, different levels of autophagy are induced in different cell types and time points. Tian et al. found that autophagosome formation in mice heads of the ischemic hemisphere peaked at 1 d after tMCAO, and became gradually weaker from 3 d after tMCAO (75). Moreover, the number of autophagy-induced neurons in the mice head of the ischemic hemisphere was about two times higher compared to the number of autophagic astrocytes at 1 d after tMCAO (75). Liu et al. found that autophagy flux in neurons remained at low levels and was activated immediately upon exposure to OGD, while astrocytes appeared the opposite (76). A series of molecular mechanisms are involved in the regulation of autophagy, including phosphatidylinositol-3-kinase/Akt-mammalian target of rapamycin (PI3k/Akt-mTOR) (28–30), Ca^2+^/5′-AMP-activated protein kinase/mTOR (Ca^2+^/AMPK/mTOR) (5, 7, 77, 78), nuclear factor kappa B/p53/mTOR (NF-κB/p53/mTOR) (79, 80), mitogen-activated protein kinase (MAPK) (81–86), and hypoxia-inducible factor 1α/BCL2 interacting protein 3 (HIF-1α/BNIP3) (7, 83, 84, 87–89) signaling pathways. These pathways are illustrated in Figure 2.

#### Maladaptive and adaptive autophagy

##### Maladaptive autophagy

###### Blood–brain barrier (BBB) disruption

The BBB is a highly organized multicellular structure composed of capillaries formed by self-fusion of brain microvascular endothelial cells (BMECs) through intact tight junctions, peripheral pericytes, and astrocytes surrounding the capillaries (90). BBB disruption occurs due to central nervous system diseases (91) and may cause secondary brain injuries, including hemorrhage and brain edema (91–94). During IS, blood flow failure causes shrinkage of BMECs, resulting in the translocation of tight junction proteins from the membrane into the cytosol. The increase in BBB permeability elevates the risk for secondary brain damage caused by I/R injury. Various mechanisms regarding the effect of autophagy on BBB during IS have been reported. Zhang et al. found that rapamycin-enhanced autophagy promoted Zonula occludens-1 (ZO-1) reduction *in vivo*, resulting in increased BBB permeability (95). In 2020, Kim et al. found that ischemia-induced maladaptive autophagy in brain endothelial cells and rat brain capillaries caused ZO-1 degradation, ultimately disrupting BBB integrity and exacerbating neurological damage. Moreover, 3-methyladenine (3-MA) inhibition of autophagy was found to reverse this adverse outcome (96). Liu et al. found that Claudin 5 (CLDN5) is delivered to the autophagosome for autophagy-lysosome-dependent degradation mediated by caveolin-1 (97). CLDN5 is a key molecule in the formation of tight junction chains in the BBB, which seals the intercellular space and maintains the paracellular barrier, and its degradation leads to BBB disruption (98). However, Li et al. recently reported the protective effects of brain endothelial cell autophagy on BBB dysfunction during cerebral I/R injury (99).

##### Cell death through apoptosis activation

Autophagy causes neural damage by triggering apoptosis. Cao et al. found that the long non-coding RNA (lncRNA) small nucleolar RNA host gene 3 (*SNHG3*) was overexpressed in the trimethylamine N-oxide (tMAO) mouse model and hypoxia-hypoglycemia/reperfusion cell model, and miR-485 was underexpressed, upregulating autophagy-related 7 (ATG7) to promote autophagy and induce neuronal apoptosis (100). Xie et al. found that neuronal ATG7 deficiency prevented basal autophagy and induced autophagy in the immature brain after hypoxia-ischemia, inhibiting neuronal apoptosis and decreasing neuronal death (101). Autophagy inhibition by 3-MA or lentivirally delivered short hairpin RNAs (shRNAs) against ATG5 and ATG7 significantly reduced the staurosporine (STS)-induced activation of caspase-3 and nuclear translocation of apoptosis-inducing factor (AIF) and provided partial protection against neuronal death (70).

##### Adaptive autophagy

Adaptive autophagy mitigates IS-related brain damage by the timely removal of old, redundant proteins and damaged organelles (7). Wang et al. found that nicotinamide phosphoribosyltransferase induces autophagy in hypoxic neuron models under OGD in a heterodimeric complex consisting of tuberin Ser1387-TOR-S6 kinase 1 (TSC2Ser1387-TOR-S6K1) signaling pathway in a silent mating type information regulation 2 homolog 1 (SIRT1)-dependent manner, promoting neuronal survival and attenuating cerebral ischemic injury (71). Carloni et al. found that rapamycin-induced autophagy exerted neuroprotective effects through PI3K/Akt-mTOR/Cyclic AMP-responsive element-binding protein (PI3K/Akt-mTOR/CREB) signaling in a young mouse model of cerebral hypoxia-ischemia (9). Ren et al. found that, in comparison with control PC12 cells, OGD/reperfusion (OGD/R) caused an increase in the miR-187-3p level and a decrease in seipin protein levels, reducing autophagic flux and enhancing apoptosis, ultimately increasing ischemia-induced cerebral damage (72). Zhang et al. found that the induction of chloride channel-3 contributed to the formation of the Beclin1 and Vps34 complex, activated autophagy, and attenuated brain ischemic injury (102). Overall, adaptive autophagy attenuates IS-induced brain damage.

#### Autophagy pathways in IS

##### MTOR signaling mediated autophagy

The mTOR is a serine/threonine protein kinase comprising rapamycin-sensitive mTOR complex 1 (mTORC1) and rapamycin-less sensitive mTOR complex 2 (mTORC2), of which mTORC1 is the primary regulatory target. mTOR kinase is a key molecule involved in autophagocytosis induction. The activation of mTOR pathways (such as the Akt and MAPK signaling pathways) inhibits autophagocytosis, whereas the negative regulation of mTOR pathways (such as the AMPK and p53 signaling pathways) promotes autophagocytosis. Hei et al. (103) found that IS induces autophagy through mTOR inhibition and alleviates the degree of cerebral ischemia in rats with ischemic injury caused by acute hyperglycemia, which may confirm the view that “moderate autophagy” may have a protective effect on “slow and mild” brain ischemic injury. Moreover, Huang et al. found that curcumin can be activated through the PI3K/Akt-MTOR pathway, alleviating the autophagic activity of nerve cells and improving cerebral IS injury in adult rats (104).

##### MAPK signaling pathway

The MAPK signaling pathway is composed of p38 extracellular regulated protein kinases (p38-ERK), ERK, and C-Jun N-terminal kinase (JNK) (105). In the early stage of stroke, activation of the p38 signaling pathway promotes Elk1, C/EBP homologous protein (CHOP10), leukocyte cell-derived chemotaxin-2 (LEF2C), and protein kinases MAPKK2/3 to maintain neuronal survival and play roles in anti-inflammatory and anti-apoptotic processes. In contrast, in the late stage of stroke, p38 MAPK is overactivated and promotes the expression of target genes activating transcription factors and caspases (106–108), promoting neuronal apoptosis; thus, differences in IS p38 MAPK signal molecules should be given different interventions. This viewpoint has been supported by relevant studies showing that rhizoma coptidis jiedu soup causes overactivation of ERK and inhibition of JNK, as well as that p38 MAPK signal induction protection autophagy is beneficial for treating IS. The Akt/Smad signaling pathway inhibits JNK and p38 MAPK molecules and negatively regulates OGD-induced autophagy in PC12 cells (73). p38 inhibitors promote cell survival signaling pathways (e.g., ERK) and attenuate ischemic mitochondrial fragmentation or autophagy, reducing ischemic cerebral infarction dead volume and protecting nerve function (86). This evidence suggests that ERK, JNK, and p38 MAPK mediate autophagy in IS. The processes by which ERK activation inhibits autophagy are contrasting to those of JNK and p38 MAPK.

##### HIF-1α/BNIP3 signaling pathway

The HIF-1α activity is correlated with ischemia-induced neuronal death. In the early stage of acute stroke, HIF-1α/HIF-2α double-knockout mice showed low expression levels of anti-survival factors, such as BNIP3, BNIP3L, and phorbol-12-myristate-13-acetate-induced protein 1 (PMAIP1), thus protecting against early acute neuronal cell death and nervous system damage (109). HIF-1α overexpression triggers mitophagy, often accompanied by inhibition of the mTOR pathway and thereby increasing neuronal survival (110). mTOR inactivation by high expression levels of HIF-1α activates AMPK and may affect the survival of bone marrow mesenchymal stem cells (BMSCs) *via* HIF-1α after transplantation (111). Transplantation of HIF-1α overexpressed BMSCs into MCAO rats reduces the cerebral infarction dead volume and improves neurobehavioral outcomes. In summation, HIF-1α may regulate AMPK and mTOR activation, leading to autophagy which may contribute to the survival of BMSCs.

## How circRNAs influence disease progression through the regulation of autophagy

Autophagy is a complex process. Emerging studies have shown that circRNAs can influence disease progression *via* autophagy regulation, by relying on the transcriptional and post-transcriptional modifications of autophagy-related genes (112). CircRNAs can regulate autophagy by influencing transcription factors at the RNA level. CircST3GAL6 promotes autophagy through FOXP2 transcriptional inhibition of the MET proto-oncogene (*MET*) to inhibit the PI3K-Akt-mTOR pathway (113). CircRNAs can regulate autophagy directly by influencing the expression of autophagy-related proteins. CircPABPN1 blocks HuR binding to Atg16l1 mRNA, thereby inhibiting autophagy by lowering ATG16L1 expression (114). CircRNAs also can regulate autophagy *via* RNA methylation. Autophagy-related circRNA (ACR) can inhibit cardiomyocyte autophagy by activating phosphatase and tensin homolog-induced putative kinase 1 (Pink1) expression *via* its blocking of the Dnmt3B-mediated DNA methylation of the Pink1 promoter. Pink1 inhibits autophagy through the phosphorylating family with sequence similarity 65 member B (FAM65B) (115). This review discusses the circRNA pathways that regulate autophagy in the following section.

### MTOR pathway

#### Akt-MTOR pathway

Xu et al. found that circST3GAL6 was downregulated in gastric cancer (GC) and was associated with poor prognosis of GC patients (113). CircST3GAL6 can upregulate forkhead box P2 (FOXP2) by sponging miR-300. FOXP2, a transcription factor, can suppress the transcriptional activity of MET to promote autophagy *via* the PI3K–Akt-mTOR pathway (113). Liu et al. found that ACR was decreased in RSC96 cells that were subjected to high glucose (HG) irritation (116). Overexpression of ACR can relieve HG-aroused RSC96 cell autophagy by promoting PI3K/AKT/mTOR pathway activation through a decrease in miR-145-3p expression (116). Wu et al. found that circ_0009910 was highly expressed in acute myeloid leukemia (AML) tissues and cells (117). Circ_0009910 can suppress autophagy by activating the PI3K/AKT signaling pathway *via* miR-491-5p/β-1, 4-galactosyltransferase 5 in AML cells (117).

#### AMPK-MTOR pathway

The AMPK pathway can induce autophagy by suppressing mTORC1. Jin et al. found that circRNA_002581 knockdown could induce autophagy to relieve injury in the methionine- and choline-deficient (MCD) diet-induced non-alcoholic steatohepatitis (NASH) mouse model (118). CircRNA_002581 can sponge miR-122 to increase cytoplasmic polyadenylation element-binding protein 1 (CPEB1) expression, which can downregulate PTEN, and then inhibit the AMPK-mTOR pathway to downregulate autophagy in the NASH-like cell model (118). Shang et al. found that circPAN3 was upregulated in bone marrow (BM) samples of AML patients and in doxorubicin (ADM)—resistant cell lines, contributing to AML drug resistance by increasing autophagy (119). CircPAN3 overexpression can upregulate autophagy through the activation of the AMPK/mTOR pathway (119). Chakraborty et al. found abrogation of circHIPK3-induced autophagy in a subset of lung cancer cell lines (120). CircHIPK3 can sponge miR124-3p to increase STAT3 expression, which can downregulate autophagy *via* the PRKAA/AMPKα/mTOR pathway in A549 cells (120).

### MAPK pathway

Yao et al. found that circEIF3K levels were decreased in inflammatory tubal epithelial cells (121). CircEIF3K can bind with MIR139-5p to increase EIF3K and BCL2 expression and MAPK/ERK phosphorylation. This activates the autophagy in inflammatory cells and finally inhibits cell vitality (121). Zhang et al. found that the expression of circ101237 gradually increased with time and promoted autophagy in the cardiomyocytes of 2-day-old mouse models of anoxia/reoxygenation (122). CircRNA_101237 can sponge let-7a-5p to promote IGF2BP3 expression (122). In turn, IGF2BP3 increases the expression of IGF2 (123), which promotes autophagy through the phosphorylation and activation of the MAPK/ERK signaling pathway (124). Jiang et al. found that circ0032821 is upregulated in patients with GC, thereby contributing to GC tumorigenesis through the inhibition of autophagy, which may result from the activation of the MAPK/ERK signaling pathway (125).

### Beclin-1 pathway

Gan et al. found that circMUC16 levels were increased in epithelial ovarian cancer tissues (126). CircMUC16 overexpression can promote cancer cell proliferation and migration by activating autophagy through the adsorption of miR199A-5p to mitigate the inhibition of BECN1 (126). Chen et al. found that circMTO1 was upregulated in cervical cancer cell lines and tumors (127). CircMTO1 can promote autophagy by adsorbing miR6893 and promoting the expression of BECN1 to enhance the invasion and migration of cervical cancer cells (127). Guo et al. found that hsa_circ_0023404 overexpression could promote autophagy by upregulating BECN1 to inhibit apoptosis and impart resistance to cisplatin (128).

### Additional pathways

Du et al. found that circDNMT1 was increased in eight different breast cancer cell lines and in patients with breast carcinoma (129). CircDNMT1 can promote the nuclear translocation of TP53, which in turn promotes the expression of autophagy-related genes to induce autophagy, thereby enhancing breast cancer progression (129). Cao et al. found that circ0009910 was upregulated in the sera of imatinib-resistant chronic myeloid leukemia patients (130). Circ0009910 can increase the expression of ULK1 by sponging miR-34a-5p. Circ0009910 also can promote the phosphorylation of ULK1 and the expression of BECN1 and LC3-II, thereby promoting autophagy to accelerate imatinib resistance (130). Ren et al. found that circZnf292 was upregulated in ischemic heart disease (131). CircZnf292 can inhibit autophagy by activating the WNT-CTNNB1 and the MTOR signaling pathways through BNIP3 inhibition, thereby reducing damage in the OGD-induced H9c2 cell line (131).

## The role of circRNAs as biomarkers and therapeutic targets in IS

Circular RNAs involved in the regulation of nerve cell development, differentiation, material transport, and axonal plasticity are highly expressed in the nervous system (15, 16). Various circRNAs participate in the pathological processes following IS. CircRNAs regulate gene expression post-transcription by competitively binding to miRNAs or RNA-binding proteins (12).

Circulating RNAs, such as Circ_0072309 (42), circular SHOC2 Leucine-Rich Repeat Scaffold Protein (circSHOC2) (24), circUCK2 (132), circCCDC9 (133), and circPHKA2 (37), have been shown to alleviate apoptotic rates and attenuate neuronal injury through miR-100/mTOR, miR-7670-3p/SIRT1, and miR-125b-5p/growth differentiation factor 11 (GDF11), suppressing the Notch receptor 1 (Notch1) signaling pathway and the miR-574-5p/superoxide dismutase 2 (SOD2) axis. Circ-forkhead box O3 (FoxO3) (23) and circRNA 0025984 (22) attenuate neuronal injury through autophagy inhibition. Circ-FoxO3 promotes brain microvessel endothelial cell (BMEC) autophagy *via* mTORC1 inhibition to clear cytotoxic aggregates. CircRNA 0025984 protects astrocytes from autophagy through the miR-143-3p/Tet methylcytosine dioxygenase 1/150-kDa oxygen-regulated protein (miR-143-3p/TET1/ORP150 pathway). CircDLGAP4 (134) overexpression decreases BBB damage and infarcts areas after stroke by inhibiting endothelial–mesenchymal transition *via* the miR-143/HECT domain E3 ubiquitin-protein ligase 1 (HECTD1) axis. CircSCMH1 (55) contributes to functional recovery after stroke by enhancing neuronal plasticity and inhibiting glial activation. Circ_0006768 (53) upregulation attenuates HBMEC injury by upregulating vascular endothelial zinc finger 1 (VEZF1) *via* miR-222-3p inhibition. Circ-Rps5 (135) decreases neuronal damage *via* the miR-124-3p/SIRT7 axis. The upregulation of circ-FoxO3, circDLGAP4, and circCCDC9 promotes the maintenance of BBB integrity.

However, some significantly upregulated circRNAs showed contrasting effects. Circ-HECTD1 (25) aggregates neuronal deficits by promoting astrocyte autophagy *via* the miR142-TIPARP axis. Circ cGLIS3 (46), circRNA TLK1 (136), circPHC3 (45), circFUNDC1 (44), and circCDC14A (43) contribute to cell death by activating apoptosis. Overexpression of circ HIPK2 (56) and circSKA3 (137) aggravates neuronal injuries.

### CircRNAs affect IS progression by regulating autophagy

#### CircHECTD1

**Figure 3:**
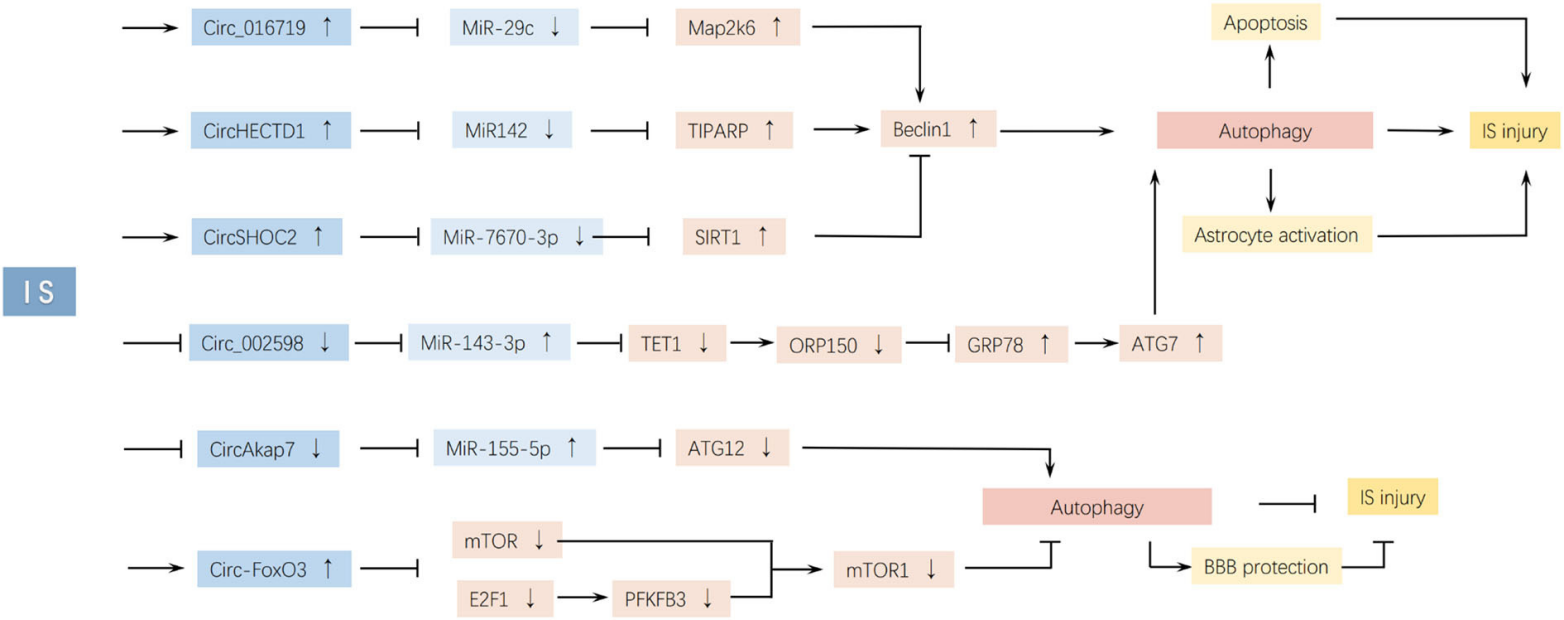
CircRNAs affect IS injury by regulating autophagy. Changing the expression of circ_016719, circHECTD1, circSHOC2, and circ_002598 can induce IS injury by upregulating autophagy. However, changing the expression of circAkap7 and circ-FoxO3 can inhibit IS injury by upregulating autophagy.

More and more studies proved that circRNAs can affect IS progression by regulating autophagy, we summarize the evidences from basic research of these related circRNAs in Table 2 and illustrate the pathways in Figure 3. The host gene of circHECTD1, HECTD1, is an E3 ubiquitin ligase that regulates cell migration machinery (138). CircHECTD1 is derived from exons 23 and 24 of *HECTD1* and is highly expressed in the brain and lungs (25). In 2018, Han et al. found the upregulation of circHECTD1 in ischemic brain tissues from a transient MCAO (tMCAO) mouse model and in plasma samples from patients with acute IS (25). Functional studies have shown reduced infarct areas, milder neuronal deficits, and less activation of astrocytes resulting from lower circHECTD1 expression in the tMCAO stroke mouse model. A mechanistic study demonstrated that circHECTD1 functions as a sponge to inhibit miR142 activity, thus upregulating TCDD-inducible poly (ADP-ribose) polymerase (TIPARP) and ultimately increasing astrocyte activation *via* macro-autophagy/autophagy (25). Therefore, downregulation of circHECTD1 expression is associated with reduced cerebral infarction.

**Table 2:** The evidence of CircRNA roles in ischemic stroke *via* autophagy regulation.

**Cell line/treatment**	**Animal/Disease model**	**Sample**	**DE CircRNA and expression**		**Regulating axis**	**Autophagy flux**	**Apoptosis** **activation**	**DE CircRNA's Roles in IS**	**References**
**Evidences from basic research**									
Primary mouse astrocyte,	Male C57BL/6J mouse	Astrocytes	circHECTD1	Up	circHECTD1/MIR142/	Upregulated	Undefined	Detrimental	(25)
A172 cell					TIPARP/Beclin1	The nucleation of autophagy			
OGD-R	tMCAO								
Primary astrocyte	Male C57BL/6J mice	Astrocytes	circSHOC2	Up	circSHOC2/miR-7670-3p/SIRT1/Beclin1	Downregulated	Decreased	Protective	(24)
						The nucleation of autophagy			
OGD	MCAO								
A172 and SK-N-AS	Male Sprague Dawley rats	Astrocytes	circ_0025984	Down	circ_0025984/miR-143-3p/TET1//ORP150/GRP78/ATG7	Upregulated	Increased	Protective	(22)
OGD	MCAO					The elongation of autophagy			
BEnd.3 or HBMEC	C57BL/6J mice	BMECs	circ-FoxO3	Up	circ-FoxO3/mTOR/mTORC1	Upregulated	Undefined	Protective	(23)
					circ-FoxO3/E2F1/PEKFB3/	The initiation of autophagy			
OGD/R	MCAO/R								
HT22 cells	Male C57BL/6J mice	Neurons	circ_016719	Up	circ_016719/miR-29c/Map2k6/	Upregulated	Increased	Detrimental	(26)
					Beclin1	The nucleation of autophagy			
OGD/R	tMCAO								
Primary astrocyte	C57BL/6 mice	Astrocytes	circAkap7	Down	circAkap7/miR-155-5p/ATG12, NRF2	Downregulated	Increased	Protective	(27)
OGD/R	tMCAO					The elongation of autophagy			

Furthermore, a recent study by He et al. found that circHECTD1 overexpression accelerated endothelial–mesenchymal transition (EndoMT) in human cerebral microvascular endothelial cells (hCMECs) treated with OGD/R, which mimics IS *in vitro* (139). As EndoMT plays a critical role in BBB dysfunction, this study indicated that increased circHECTD1 expression might damage BBB integrity (140). In addition to IS, circHECTD1 participates in the regulation of various pathological processes and diseases, including acute lung injury (141), hypertrophic scar fibrosis (142), ulcerative colitis (143), gastric cancer (144), hepatocellular carcinoma (145), and glioma (146). CircHECTD1 overexpression reduced colonic injury and inflammation by promoting autophagy in lipopolysaccharide (LPS)-induced Caco-2 cells in dextran sodium sulfate (DSS)-treated mice (143). CircHECTD1 knockdown upregulates HECTD1 protein expression in normal human pulmonary fibroblasts (HPF-a) cells and those exposed to silicon dioxide (SiO_2_). HECTD1 promotes fibroblast activation and migration *via* autophagy, promoting pulmonary fibrosis progression (147).

#### CircSHOC2

CircSHOC2 (circ_0092670) is transcribed from the SHOC2 gene and shows significantly increased expression in ischemic preconditioning astrocyte exosomes (IPAS-EXOs). SHOC2 is a prototypical leucine-rich repeat protein that promotes downstream receptor tyrosine kinase (RTK)/RAS signaling and plays important roles in several cellular and developmental processes (148). In 2020, Chen et al. found that exosomes from ischemia-preconditioned astrocytes inhibited ischemia-induced neuronal apoptosis by downregulating autophagy. The authors further validated that circSHOC2 in these exosomes reduced OGD/R-induced neuronal death. CircSHOC2 suppresses neuronal apoptosis and ameliorates neuronal damage by inhibiting autophagy and acting on the miR-7670-3p/SIRT1 axis (24). CircSHOC2 is highly expressed in IPAS-EXOs but not in neurons. It acts as a sponge for miR-7670-3p without regulating miR-7670-3p expression levels, thereby promoting SIRT1 expression to reduce astrocyte autophagy, decrease apoptosis, and protect neurons from ischemia-induced damage (24). CircSHOC2 overexpression inhibits Gag-LC3 accumulation, LC3-I-to-LC3-II conversion, and translocase of inner mitochondrial membrane 23 (TIMM23) degradation in neurons. The protective effects of circSHOC2 are abolished by an autophagic inhibitor (24). Exosomes function locally or can be stably transferred to recipient cells (149, 150). With a diameter of 30–100 nm, exosomes can cross the BBB (151). Therefore, exosomes with circRNAs are new potential therapeutic agents for tissue recovery after IS.

#### Circ_0025984

Zhou et al. found that circ_0025984 (hsa_circ_0025984, parent gene SLC38A2) and ten-eleven translocation methylcytosine dioxygenase 1 (TET1) were significantly downregulated, and miR-143-3p was notably upregulated in the tMCAO rat model, which was further demonstrated in astrocytes undergoing OGD/R. The abnormally low expression of circ_0025984 increased astrocyte apoptosis and autophagy through the miR-143-3p/TET1/ORP150 pathway, ultimately causing ischemic cerebral injury. Circ_0025984 overexpression functions as a sponge for miR-143-3p and upregulates TET1, inducing 150-kDa oxygen-regulated protein (ORP150) expression, which decreases glucose-regulated protein 78 kDa (GRP78) levels. GRP78 also induces ATG7 expression (22), promoting autophagy activation. TET1, a member of the Tet family, can convert 5-methylcytosine to 5-hydroxymethylcytosine in a 2-oxoglutarate- and Fe(II)-dependent manner and is involved in DNA demethylation (152). Another study showed that TET1 could also activate the transcriptional expression of glucose metabolism-related genes in hepatocytes (153). TET1 plays a protective role in cells undergoing OGD and induces ORP150 (an ER-associated chapel) expression by binding to its promoter DNA and consequently decreasing its methylation (22). The ORP150, a member of the heat shock protein family, is located in the endoplasmic reticulum (ER), functions as a molecular chaperone in the transport and folding of newly synthesized proteins (154), and prevents ischemia-induced cell death. Its upregulation modulates ATP/ADP exchange in GRP78 and decreases GRP78 expression (a calcium-dependent protein induced to maintain ER and cell homeostasis during calcium dysregulation, oxygen and glucose deprivation, or inflammation), ultimately maintaining calcium homeostasis and inhibiting autophagy and apoptosis (155). GRP78 induces ATG7 expression after OGD (22), thus enabling the transition of LC3-I to LC3-II, subsequently activating autophagy (156), inhibiting aggregation, and promoting the degradation of misfolded proteins (157). Therefore, circ_0025984 overexpression ultimately inhibits elongation and phagosome formation of autophagy (7), decreases apoptosis of astrocytes, and alleviates cerebral injury in MCAO rats.

By regulating autophagy, MiR-143 is involved in many diseases, such as myocardial I/R injury (158–160), Crohn's disease (161), pancreatic cancer (162, 163), acute myeloid leukemia (164), renal cell cancer (165), small-cell lung cancer (166) endometriosis (167), and prostate cancer (168).

#### Circ-FoxO3

In 2022, Yang et al. found that circ-FoxO3 (hsa_circ_0006404, parent gene FOXO3) alleviated BBB damage by activating autophagy in BMECs and in MCAO/R mice models. They found that circ-FoxO3 was upregulated in brain tissues after I/R injury, especially in BMECs and astrocytes. Furthermore, circ-FoxO3 protected against OGD/R-stimulated endothelial barrier damage and tMCAO-induced BBB collapse in mice by upregulating autophagy (23). Mechanistically, circ-FoxO3 plays a role in the sequestration of mTOR and E2F Transcription factor 1 (E2F1) to inhibit mTORC1 activity, thereby activating autophagy (23). This interaction was reported by Du et al. in 2017, who found that circ-FoxO3 could competitively sequester mTOR and inhibit mTORC1 activity, interact with E2F1 in the cytosol, block E2F1 entrance into the nucleus, and prevent its transcriptional regulation (169). The translocation of mTORC1 to lysosomes is blocked, and mTORC1 is inactivated. Finally, circ-FoxO3 promotes initiation autophagy (23).

Circ-FoxO3 was first reported to have a significant effect on modulating cell proliferation, migration, invasion, and apoptosis through different signaling pathways (170). Ectopic expression of circ-Foxo3 promoted cellular senescence, whereas silencing of circ-Foxo3 decreased cell senescence and apoptosis (169). Circ-Foxo3 is involved in the development and tumorigenesis of many cancers. Circ-Foxo3 is significantly downregulated in esophageal squamous cell cancer, bladder cancer, colorectal cancer, and acute lymphocytic leukemia (171–175). Mechanistically, circ-FoxO3 decreases the interaction between FoxO3 and MDM2 Proto-Oncogene (MDM2), and represses the poly-ubiquitination of FoxO3 modulated by MDM2, which increases FoxO3 activity, promoting p53 upregulated modulator of apoptosis (PUMA) expression and cell apoptosis (176). Circ-FoxO3 retards cell cycle progression by binding to p21 and cyclin-dependent kinase 2 (CDK2). CDK2 initiates the G1-S phase transition by binding to cyclin E (177), which can be stimulated by binding between p21 and CDK2 (178). Circ-FoxO3 overexpression promotes the formation of the circ-FoxO3–p21–CDK2 ternary complex, which hijacks CDK2 together with p21 to avoid the formation of the cyclin E/CDK2 complex, thus blocking the G1-S phase transition and the progression of the cell cycle in the S phase (179).

#### Circ_016719

In 2019, Tang et al. found that circ_016719 could activate autophagy to partly inhibit cell viability in HT22 cells and mouse models with MCAO/R (26). Circ_016719 is upregulated in brain tissues after I/R injury. Results of mechanistic studies demonstrated that circ_016719 directly sponges miR-29c to increase the expression of Rac-MAPK kinase 6 (Map2k6) (26). Map2k6, also known as MKK6, is a critical upstream regulator of the MAPK pathway, with key roles in cell survival, differentiation, and inflammation (180). Circ_016719 may activate autophagy to promote nucleation *via* the upregulation of beclin-1 and p53.

#### CircAkap7

In 2017, Mehta et al. found that circAkap7 is downregulated in tMCAO mice compared to sham mice (181). CircAkap7 (mmu_circ_0000154, mm9_circ_010383) is derived from exon 2 of the Akap7 gene, located on chromosome 10 (1024987113-25009536), whose spliced mature sequence length is 579 bp (27). In 2020, Xu et al. found that exo-circAkap7 treatment decreased tMCAO-induced cerebral inflammation, apoptosis, and oxidative stress, and promoted autophagy. Further mechanistic research demonstrated that circAkap7 directly sponges miR-155-5p to increase the expression of NRF2 and ATG12 in OGD-treated astrocytes to promote autophagy and ameliorate oxidative stress, suggesting that the use of exo-circAkap7 is a potential treatment strategy for cerebral ischemic injury (27). ATG12 is indispensable for the formation of ATG5–ATG12–ATG16L1 complex, which supports the elongation of autophagosomes (182).

#### Hsa_Circ_0001599

In 2021, Li et al. found that hsa_circ_0001599 was significantly upregulated in patients with large-artery atherosclerotic stroke, and its level positively correlated with stroke neurological severity and infarct volume (183). The parental gene of hsa_circ_0001599, FK506-binding protein 5 (FKBP5), is a member of the immunophilin protein family and plays a role in stress response and inflammation (184). Yu et al. found that FKBP5 was upregulated in patients with acute IS, and it regulated autophagy through the downstream AKT/FoxO3 signaling pathway in the OGD/R cell model (185). Therefore, hsa_circ_0001599 may be a pivotal molecule affecting autophagy after IS.

## Conclusion

Stroke is widely known for its high mortality, disability, and recurrence rates, and IS accounts for the majority of the cases. Ischemia and hypoxia activate many pathological processes, such as apoptosis, inflammation, oxidative stress, angiogenesis, and autophagy, among others. CircRNAs have been shown to be differentially expressed during IS and to be participating in all the above processes. One circRNA can influence more than one process in IS, including autophagy, which has become the focus of recent research. However, previous studies have not yielded consistent results. Activated autophagy aggravates neurological dysfunction by exacerbating neuronal death through apoptosis activation and BBB disruption or decreases brain damage by reducing apoptosis. Astrocytes are excellent candidates for regulation in autophagy activation to protect the brain against IS-induced injury owing to their numerical advantages and importance in cerebral parenchymal homeostasis and normal brain function. CircRNAs are differentially expressed during IS and are involved in the regulation of IS injury. CircRNAs influence IS progression, mostly by sponging miRNAs. Four circRNAs have been found to be differentially expressed in astrocytes and influence IS progression by regulating autophagy. Moreover, one circRNA can regulate autophagy through different pathways. These studies provide a new potential treatment for IS through autophagy regulation by altering circRNA expression. However, unresolved questions remain. First, autophagy activation is either a neuroprotective process or a cause of cell death and remains controversial. Second, one circRNA can sponge more than one miRNA, influence many other processed cells, and may contribute to other diseases. Many circRNAs are differentially expressed in IS, and one circRNA can regulate autophagy through different pathways; therefore, determining whether one or more circRNAs could be selected as treatment targets for IS still requires further research. Furthermore, the transportation of the target circRNAs or autophagy regulators into the brain is yet to be fully elucidated. Furthermore, if all circRNAs are altered during IS, the protective and/or detrimental role may be enhanced. Accordingly, more research is required in this direction. Finally, because of the narrow therapeutic time window, blocking the mechanism of neuronal death in the ischemic cascade could be difficult.

## Author contributions

XL, LL, and LJ contributed to the review design and revised the manuscript. XL drafted the manuscript. LJ and LL revised the manuscript. All the authors have read and approved the final version of the manuscript.

## Funding

This work was funded by Zhejiang Basic Public Welfare Research Program (LGF20H090008 and GC22H095676) and Hangzhou Health Science and Technology Project (ZD20200056, ZD20210007, and A20200341).

## Conflict of interest

The authors declare that the research was conducted in the absence of any commercial or financial relationships that could be construed as a potential conflict of interest.

## Publisher's note

All claims expressed in this article are solely those of the authors and do not necessarily represent those of their affiliated organizations, or those of the publisher, the editors and the reviewers. Any product that may be evaluated in this article, or claim that may be made by its manufacturer, is not guaranteed or endorsed by the publisher.

## Abbreviations

IS: Ischemic stroke
circRNAs: circular RNAs
RBPs: RNA-binding proteins
miRNAs: microRNAs
I/R: ischemia/reperfusion
HIF-1α: hypoxia-inducible factor 1α
mTORC1: mTOR complex 1
mTOR: mammalian target of rapamycin
AMPK: 5′-AMP-activated protein kinase
LKB1: liver kinase B1
CAMKKβ: calcium/calmodulin-dependent protein kinase kinase 2
BNIP3: Bcl-2/adenovirus E1B 19 kDa protein-interacting protein 3
JNK: c-Jun NH2-terminal kinase
tMCAO: transient middle cerebral artery occlusion
pMCAO: permanent middle cerebral artery occlusion
OGD/R: oxygen-glucose deprivation/reoxygenation
3-MA: 3-methyladenine
PTH: Pien Tze Huang
BMECs: brain microvascular endothelial cells
CLDN5: claudin 5
BBB: blood–brain barrier
SIRT1: silent mating type information regulation 2 homolog 1
TIPARP: TCDD-inducible poly [ADP-ribose] polymerase
TET1: Ten-eleven translocation methylcytosine dioxygenase 1
ER: endoplasmic reticulum
ORP150: 150-kDa oxygen-regulated protein
LAA: large-artery atherosclerotic
FKBP5: FK506-binding protein 5.
